# Potential Cytoprotective and Regulatory Effects of Ergothioneine on Gene Expression of Proteins Involved in Erythroid Adaptation Mechanisms and Redox Pathways in K562 Cells

**DOI:** 10.3390/genes13122368

**Published:** 2022-12-15

**Authors:** Victoria Simões Bernardo, Flaviene Felix Torres, Carla Peres de Paula, João Pedro Maia de Oliveira da Silva, Eduardo Alves de Almeida, Anderson Ferreira da Cunha, Danilo Grünig Humberto da Silva

**Affiliations:** 1 Department of Biology, Universidade Estadual Paulista (UNESP), José do Rio Preto 15054-000, SP, Brazil; 2 Department of Genetics and Evolution, Universidade Federal de São Carlos (UFSCar), São Carlos 13565-905, SP, Brazil; 3 Department of Natural Sciences, Fundação Universidade Regional de Blumenau (FURB), Blumenau 89030-000, SC, Brazil; 4 Campus de Três Lagoas, Universidade Federal de Mato Grosso do Sul (CPTL/UFMS), Três Lagoas 79613-000, MS, Brazil

**Keywords:** antioxidants, therapeutic agent, redox adaptation, proteasome

## Abstract

This study aimed to establish the importance of ergothioneine (ERT) in the erythroid adaptation mechanisms by appraising the expression levels of redox-related genes associated with the PI3K/AKT/FoxO3 and Nrf2-ARE pathways using K562 cells induced to erythroid differentiation and H_2_O_2_-oxidative stress. Cell viability and gene expression were evaluated. Two concentrations of ERT were assessed, 1 nM (C1) and 100 µM (C2), with and without stress induction (100 µM H_2_O_2_). Assessments were made in three periods of the cellular differentiation process (D0, D2, and D4). The C1 treatment promoted the induction of *FOXO3* (D0 and 2), *PSMB5*, and *6* expressions (D4); C1 + H_2_O_2_ treatment showed the highest levels of *NRF2* transcripts, *KEAP1* (D0), *YWHAQ* (D2 and 4), *PSMB5* (D2) and *PSMB6* (D4); and C2 + H_2_O_2_ (D2) an increase in *FOXO3* and *MST1* expression, with a decrease of *YWHAQ* and *NRF2* was observed. in C2 + H_2_O_2_ (D2) an increase in *FOXO3* and *MST1*, with a decrease in *YWHAQ* and *NRF2* was observed All ERT treatments increased gamma-globin expression. Statistical multivariate analyzes highlighted that the Nrf2-ARE pathway presented a greater contribution in the production of *PRDX1*, *SOD1*, *CAT*, and *PSBM5* mRNAs, whereas the PI3K/AKT/FoxO3 pathway was associated with the *PRDX2* and *TRX* transcripts. In conclusion, ERT presented a cytoprotective action through Nrf2 and FoxO3, with the latter seeming to contribute to erythroid proliferation/differentiation.

## 1. Introduction

One of the most critical features in human physiology is redox homeostasis, playing a pivotal role in physiological and pathophysiological situations [1]. This biological redox equilibrium influences several cell signaling pathways, a common pathophysiological mechanism that underlies a wide range of chronic and degenerative diseases such as immunological, neurodegenerative, cardiovascular, oncological, hemolytic disorders, and age-related diseases [2,3,4,5,6]. The accumulation of oxidative damage presents an intrinsic relationship with the severity of these diseases [7,8], which leads to the assumption that antioxidant therapies represent a promising path for alternative treatment. However, the simple administration of exogenous antioxidants did not have the expected benefits [9,10,11]. Therefore, another option that has been explored involves compounds capable of inducing the expression of transcription factors in the redox code. Thus, acting in the modulation of signaling pathways that play central roles in controlling the expression of antioxidant defense enzymes, such as Nrf2 and FoxO3 [12,13].

Under physiological conditions, Nrf2 (nuclear factor erythroid 2-related factor 2) is degraded through the ubiquitin-proteasome pathway, a mechanism in which the Keap1 (Kelch-like ECH-associated protein 1) oxidative stress sensor serves as an adapter for the Cul3-Rbx ubiquitin ligase complex. In the Nrf2 activation mechanism (under oxidative stress), the Keap1-Cul3-Rbx1 complex disconnects from Nrf2, allowing nuclear translocation and activation of the Antioxidant Response Element (ARE) promoter. This pathway initiates the transcription of numerous antioxidants, such as superoxide dismutase 1 (*SOD1*), catalase (*CAT*), glutathione peroxidase 1 (*GPX1*), peroxiredoxins 1, 2, 5, and 6 (*PRDX1*, *2*, *5* and *6*) [14,15], as well as stimulating Fetal Hemoglobin (HbF) [16], and proteasome subunit beta type 5 and 6 (*PSMB5* and *6*) transcription [17,18].

In contrast, FoxO3 (Forkhead box class O3) has its activity regulated mainly through post-translational modifications in response to external stimuli. Under the stimulus of growth factor, for example, this transcription factor is negatively regulated through phosphorylation via the PI3K/AKT (phoinositide 3-kinase/protein kinase B) signaling pathway (translocation mediated by chaperone 14-3-3, human gene symbol *YWHAQ*), thus preventing the transcription of its target genes [19,20,21]. When under oxidative stress, the MST1 (mammalian sterile 20-like 1) kinase phosphorylates the cytoplasmic FoxO3 (blocking the interaction with 14-3-3), promoting its nuclear translocation. Thus, through the transcription of its target genes, FoxO3 controls whether the cell enters the path of survival (by inducing antioxidants genes, such as *SOD1* and *2*, *CAT*, *GPX1*, *TRX1*, and *PRDX1*, *2*, *3*, and *5*) or apoptosis (pro-apoptotic and CDK inhibitors genes), in an intensity-dependent manner [19,20,21].

Regarding potential therapeutic agents, the dietary antioxidant *L*-ergothioneine (ERT, 2-mercaptohistidine trimethylbetaine) has been recently highlighted in a wide range of studies. This low-molecular-mass thiol-based antioxidant synthesized by microorganisms [22] is associated with health and disease and the mechanisms of cellular and tissue protection (revised in detail in [23]). This natural antioxidant presents a unique chemistry that gives rise to its high stability and ability to accumulate in the body [24], enabling a wide range of cytoprotective, disease-mitigating properties [25] and lacking toxicity or adverse effects associated with its administration [26,27,28,29].

In addition to these unique properties, evidence suggests that ERT may act indirectly in maintaining thiol levels in vivo [30], modulating the activation of redox pathways, such as Nrf2-ARE, to protect cells against oxidative stress [31]. Furthermore, the high expression of the ERT-specific transporter (ETT) in hematopoietic precursor cells, maturing erythroid cells that produce high levels of hemoglobin, and even the tissues for the production of these cells (fetal liver or bone marrow) relative to other major tissues [32,33], corroborate the hypothesis of a critical role of this nutraceutical during hematopoiesis and in determining the health of mature red blood cells. Such observations support the notion that ERT has enormous potential for therapeutic intervention and is a potentially essential dietary micronutrient associated with the processes of differentiation and proliferation of erythroid cells.

However, despite its importance in the animal diet, its mechanisms of action still need to be elucidated. In this regard, for the first time in the literature, we propose to investigate ERT’s role in controlling transcriptional patterns related to the main redox signaling pathways in erythroid cells. We believe that its concentration at very low levels, like other important erythropoiesis regulators, such as erythropoietin (plasma concentration of ~5 pmol/l [34]), is fundamental for the complex and multiple-step regulation of erythroid cells. Thus, we hypothesized that this dietary antioxidant could play relevant roles in the erythroid adaptation mechanisms at different stages of K562 cell maturation by appraising the expression levels of several redox-related genes associated with the PI3K/AKT/FoxO3 and Nrf2-ARE pathways. In other words, gene regulation occurs in response to an increase in the oxidative load, which, in turn, shifts the cells to a low-intensity oxidative stress state and the induction of the redox-dependent response.

## 2. Materials and Methods

### 2.1. Cell Culture

K562 cells (chronic myelogenous leukemia-ATCC; catalog number CCL-243) were maintained in RPMI-1640 (Roswell Park Memorial Institute, Buffalo, NY, USA), with 10% Bovine Fetal Serum (BFS, Hyclone, Logan, UT, USA), 100 U/mL penicillin, 100 μg/mL streptomycin, and 0.25 μg/mL fungicide amphotericin B in an incubator at 37 °C under a humidified atmosphere of 5% CO_2_. The cells were treated with 50 μM of hemin and 100 μM of hydroxyurea, following the protocol previously established, to induce erythroid differentiation in the K562 cell line [35]. This study was approved by the Data Safety Monitoring Board (DSMB) according to Brazilian Regulations.

### 2.2. Identification of Erythroid Differentiation

The erythroid differentiation of K562 cells was assessed by a benzidine cytochemical test [36]. A total of 50 µL of 30% hydrogen peroxide mixed with 10 µL of benzidine solution was added to 100 µL of cell suspension. The mixture was incubated in the dark at room temperature for 25 min, and the percentage of benzidine-positive cells was determined by microscopic examination. Gamma (γ)–globin mRNA was detected by the RT-PCR method, and the mRNA levels were also used as an indicator of erythroid differentiation.

### 2.3. Viability

Cell viability was assessed using the Trypan blue (Sigma-Aldrich, St. Louis, MO, USA) exclusion assay in the final concentration of 0.1%. The ratio between the number of living (L) and dead (D) cells provided the percentage (L/D × 100) of cell viability [37].

### 2.4. Experimental Design

The study had three pseudo-replicated samples followed during five days of cell differentiation, with collections of biological samples in three periods of the differentiation process: before the start (D0), at the beginning of differentiation (D2), and the end of the process (D4), with the prediction transcripts and protein level analyses, as well enzyme activities of the biomarkers proposed. On day 0 of the experiment, 3.1 × 10^6^ cells/mL (100% viable and 70–80% confluent) were placed in polystyrene bottles in a final volume of 20 mL of culture medium, induced to erythroid differentiation through the addition of hemin (50 μM) and hydroxyurea (100 μM) and incubated at 37 °C/5% CO_2_. In addition, each sample was individually divided into the following groups: erythroid cells without inducing oxidative stress or ERT treatment (Reference); cells under stress induction with hydrogen peroxide (100 µM H_2_O_2_); cells treated with a low concentration (C1) of ERT 1 nM and a high concentration (C2) of ERT 100 µM; and finally, two sets of cells treated with the same concentrations of ERT associated with stress induction (C1 + H_2_O_2_ and C2 + H_2_O_2_, respectively).

### 2.5. Administration of Ergothioneine (Protective Agent) and Hydrogen Peroxide (Stressing Agent)

**Figure 1:**
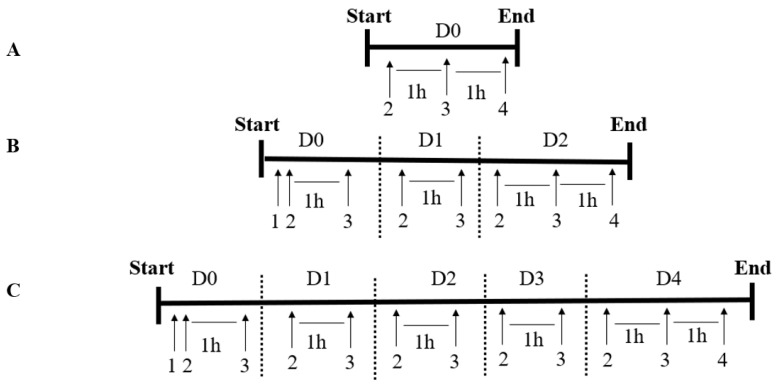
Experimental Design. (**A**)—Summary of the experimental design for the treatments evaluated at D0 (day 0). (**B**)—Summary of the experimental design for the treatments evaluated on D2 (day 2). (**C**)—Summary of the experimental design for the treatments evaluated on D4 (day 4). D0: before the differentiation process; D2: the beginning of cell differentiation, with increased hemoglobin F (HbF) expression; and D4: maximum of the differentiation process, with the peak of HbF expression and increase in cell volume along with cellular complexity and the presence of cells with different shapes can be observed (pear-shaped, circular, cells with vesicles, among other shapes). (1) Induction of erythroid differentiation with hemin and hydroxyurea administration. (2) Ergothioneine treatments (1 nM or 100 µM). (3) Stressor agent administration (100 µM H_2_O_2_). (4) Biological material separation.

The concentration adopted of 100 μM H_2_O_2_ had been previously tested and validated by de Paula (2020) for this experimental model [35]. For the ERT treatments, we adopted 1 nM as the low dose since the pM-nM scales are usually observed in regulatory molecules, such as EPO and others, as well as based on some of our previous studies with a different antioxidant, which was melatonin [34,38]. For the high dose (100 µM), we used a therapeutic one, according to the levels found in the plasma (~400 nM) [39] and erythrocytes (~50 µM) [40] of healthy individuals and its well-tolerance and low toxicity [27,29]. The administrations of the antioxidant and peroxide were carried out daily and in a staggered manner in the associated groups (C1 + H_2_O_2_ and C2 + H_2_O_2_); that is, one hour after the administration of the respective concentrations of ERT, H_2_O_2_ was added. The samples were collected 1 h after the administration of the stressor, on days 0 (D0), 2 (D2), and 4 (D4). The experimental procedure is summarized in Figure 1.

### 2.6. Real-Time PCR

RNA samples were subjected to DNAseI treatment (Invitrogen, Rockville, MD, USA) and reverse transcribed with a *High-Capacity cDNA Reverse Transcription kit* (Thermo Scientific, Waltham, MA, USA) using oligo dTV and random primers blend. The sequence of primers is shown in Table S1 in the supplementary material. The concentration of each primer was determined, and the amplification efficiency was calculated according to the equation E^(1/slope)^ to confirm the accuracy and reproducibility of the reactions. Amplification specificity was by the dissociation protocol. qPCRs were performed in a *StepOne Plus Real-time PCR System* (Thermo Scientific, Waltham, MA, USA), using *SYBR Green GoTaq Master Mix* (Promega, Madison, WI, USA). The fold change in mRNA level was calculated using 2^−ΔΔCt^ [41], and all the values were normalized to the expression of the beta-actin (ACTB) gene.

### 2.7. Statistical Analyses

Univariate analyzes were performed using the Statistica 9.0 software (Statsoft Inc., Tulsa, OK, USA), while the graphs were made using the GraphPad Prisma software version 5.01 for Windows (GraphPad Software, La Jolla, CA, USA). Data normality was verified using Normal Probability Plots of Residuals. Thus, some data were transformed by log10 when necessary. For comparison between groups, General Linear Models (GLM) were adopted in the ANOVA two-ways design, allowing to verify the effects of treatments, incubation periods, as well as any interactions between these predictors on the dependent variables. Each experiment was analyzed relative to its own reference group [42]. As a multivariate alternative for analyzing the degree of association, the General Regression Model (GRM) analysis with multiple regression design was adopted, implementing more about the relationship between several independent variables and a dependent one. This analysis also provided the partial correlation, that is, the individual contribution of a certain independent variable from the set on the dependent one, after the control for all the other variables in the equation [43]. The results were expressed as mean ± SEM of their biological values, and all statistical analyses were considered significant at *p* < 0.05.

## 3. Results

### 3.1. Cell Viability

Contrary to expectations, no significant deleterious effect was observed in K562 cells subjected to oxidative stress induction with H_2_O_2_, making it impossible to assess possible protective effects of ERT treatments on cell viability in groups C1 + 100 µM H_2_O_2_ and C2 + 100 µM H_2_O_2_ (Figure S1–Supplementary material). On the other hand, it is worth noting that treatments with ERT (C1 and C2) did not present toxicity with compromised viability of K562 cells in any of the differentiation periods. Thus, viability ranged from 77.7% (D4–100 µM H_2_O_2_) to 100% (D0–all treatments) in all experimental situations tested.

### 3.2. Erythroid Differentiation

**Figure 2:**
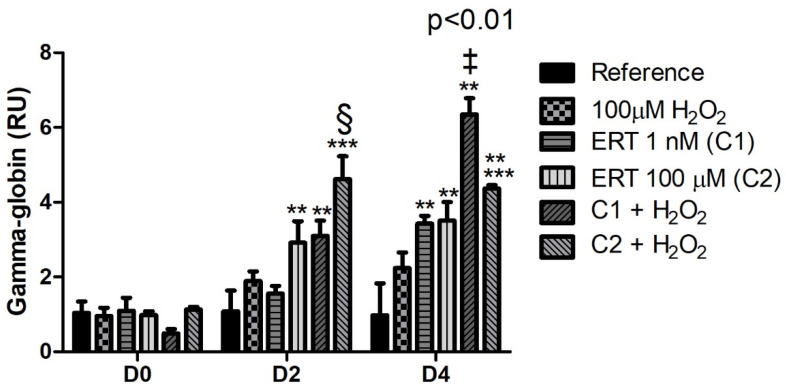
Relative expression of Gamma-Globin in K562 erythroid cells. Reference: K562 cells without H_2_O_2_ and not treated with ERT; 100 µM H_2_O_2_: Cells under stress induction with hydrogen peroxide; C1: cells translated with 1 nM ergothioneine (ERT); C2: cells translated with 100 µM ERT; C1 + 100 µM H_2_O_2_ and C2 + 100 µM H_2_O_2_: sets of cells treated with the same concentrations of ERT associated with stress induction; D0: before the differentiation process, D2: beginning of cell differentiation; D4: end of the process; and RU: Relative Unit-fold-change of the transcripts related to the Reference subgroup in D0. The symbols indicate the following statistical differences: § Effect of the higher concentration of ERT (C2) associated with 100 µM H_2_O_2_ compared to treatment C2; ‡ Effect of ERT C1 + 100 µM H_2_O_2_ treatment compared to all treatments; ** Effect of treatment within each period, compared to treatment Reference; and *** Effect of treatment within each period, compared to treatment 100 µM H_2_O_2_. The representative values of the three pseudoreplicates were expressed as mean with standard error (±SEM). General Linear Models (GLM) with two-way ANOVA format, complemented by the Bonferroni test.

The comparisons of the relative gene expression of mRNA γ-globin (Figure 2) showed an increased expression during the erythroid differentiation process (days 2 and 4), with higher transcript levels (up to ~6.5-fold increase) at the end of the process (D4). Additionally, it is interesting to notice that almost all ERT treatments, regardless of hydrogen peroxide administration, showed higher mRNA levels than their respective controls during the erythroid differentiation process. Such observations suggest that ERT may influence this gene expression.

### 3.3. Keap1/Nrf2/ARE Signaling Pathway

**Figure 3:**
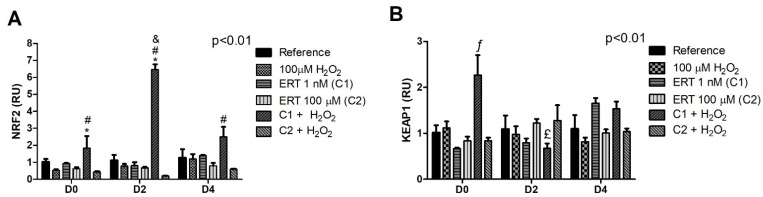
Relative expression of the Keap1/Nrf2/ARE pathway in K562 erythroid cells. (**A**)—*Nrf2* gene expression. (**B**)—*Keap1* gene expression. Reference: K562 cells without H_2_O_2_ and not treated with ERT; 100 µM H_2_O_2_: Cells under stress induction with hydrogen peroxide; C1: cells translated with 1 nM ergothioneine (ERT); C2: cells translated with 100 µM ERT; C1 + 100 µM H_2_O_2_ and C2 + 100 µM H_2_O_2_: sets of cells treated with the same concentrations of ERT associated with stress induction; D0: before the differentiation process; D2: the beginning of cell differentiation; D4: end of the process; and RU: Relative Unit-fold-change of the transcripts related to the Reference subgroup in D0. The symbols indicate the following statistical differences: * Effect of treatment C1 + 100 µM H_2_O_2_ within each period; # Effect of the differentiation period (D2) compared to the same treatment (C1 + 100 µM H_2_O_2_) in the other differentiation periods; & Effect of lower concentration of ERT (C1) compared to higher (C2), both associated with 100 µM H_2_O_2_; ƒ Increased levels of transcripts in the C1 + 100 µM H_2_O_2_ group compared to the others within the D0 period (except for the 100 µM H_2_O_2_ group); and £ Effect of the differentiation period (D2) compared to the same treatment (C1 + 100 µM H_2_O_2_) only on D0. The representative values of the three pseudoreplicates were expressed as mean with standard error (± SEM). General Linear Models (GLM) with two-way ANOVA format, complemented by the Bonferroni test.

The comparisons of the relative gene expression of the Keap1/Nrf2/ARE signaling pathway showed a 6.5-fold increase of *Nrf2* levels in C1 + H_2_O_2_ (Figure 3A). Furthermore, a peak of mRNA expression of *Keap1* (~2-fold increase) can be observed in C1 + H_2_O_2_ on D0 (Figure 3B). These results reveal an intriguing effect of the treatment (C1 + H_2_O_2_) within the differentiation periods; the effect of the lower concentration of ERT (C1) compared to the highest (C2), both associated with the addition of H_2_O_2_ within each evaluated period. In addition, in the same treatment (C1 + H_2_O_2_), there is a significant reduction (~1.5-fold) in the transcript levels of *Keap1* at the beginning of the differentiation process (D2) in relation to the same treatment in D0. A similar pattern (although not statistically significant) can be observed on day 4. Such observations reinforce the cytoprotective effect of ERT when cells are in the presence of H_2_O_2_, suggesting a conditioned action of ERT.

### 3.4. FOXO3 and Its Subcellular Location Regulators

**Figure 4:**
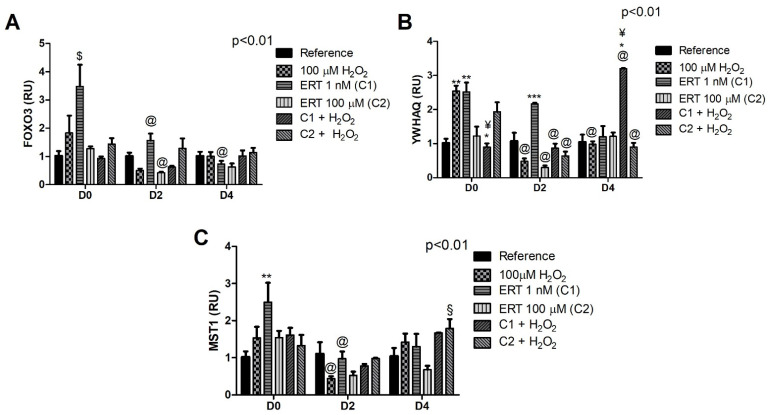
Relative expression of FOXO3 pathway in K562 erythroid cells. (**A**)—*FOXO3* gene expression. (**B**)—*YWHAQ* (14-3-3) gene expression. (**C**)—*MST1* gene expression. Reference: K562 cells without H_2_O_2_ and not treated with ERT; 100 µM H_2_O_2_: Cells under stress induction with hydrogen peroxide; C1: cells translated with 1 nM ergothioneine (ERT); C2: cells translated with 100 µM ERT; C1 + 100 µM H_2_O_2_ and C2 + 100 µM H_2_O_2_: sets of cells treated with the same concentrations of ERT associated with stress induction; D0: before the differentiation process; D2: the beginning of cell differentiation; D4: end of the process; and RU: Relative Unit-fold-change of the transcripts related to the Reference subgroup in D0. The symbols indicate the following statistical differences: $ Effect of the lowest concentration of ERT (C1) within each period; ¥ Effect of lower concentration of ERT (C1) compared to higher (C2), both associated with 100 µM H_2_O_2_; § Effect of the higher concentration of ERT (C2) associated with 100 µM H_2_O_2_ compared to treatment C2; @ Effect of the differentiation period within each treatment, compared to its counterpart in D0; * Effect of treatment C1 + 100 µM H_2_O_2_ within each period, compared to the treatments 100 µM H_2_O_2_ and ERT C1; ** Effect of treatment within each period, compared to the Reference; and *** Effect of treatment within each period, compared to treatment 100 µM H_2_O_2_. The representative values of the three pseudoreplicates were expressed as mean with standard error (± SEM). General Linear Models (GLM) with two-way ANOVA format, complemented by the Bonferroni test.

The treatment with the lowest concentration of ERT (C1) promoted the induction of *FOXO3* expression (~3.5-fold increase) when compared to the other treatments in the same differentiation period (D0) and, interestingly, only in this period. The highest concentration tested (C2) promoted a ~2.5-fold reduction in the levels of transcripts at the beginning of the differentiation process (D2), remaining low until the end of the experiment (Figure 4A). Although not statistically significant, a pattern of increased expression of this transcription factor can be observed in the ERT C2 + 100 µM H_2_O_2_ when compared to ERT C1 + 100 µM H_2_O_2_ (D0 and 2).

In relation to the *YWHAQ* gene (Figure 4B), C1 + 100 µM H_2_O_2_ seems to induce the expression during the cell differentiation process, while the opposite behavior is observed for C2 + 100 µM H_2_O_2_. Additionally, a ~2.5-fold increase of gene expression was observed in the Peroxide and ERT C1 treatments (D0) when compared to the reference, demonstrating an effect of these treatments that is no longer observed in the other evaluated periods. Finally, when observing the expression of the *MST1* kinase gene (Figure 4C), a decrease in the expression of this kinase was observed in the peroxide and ERT C1 treatments (D2). These expression profiles allude to MST1-FoxO3 pathway activation (increase in *FOXO3* and *MST1*, concomitantly with a decrease in 14-3-3), which suggests a possible cytoprotective effect of ERT for the ERT C2 + H_2_O_2_ treatment.

### 3.5. Gene Expression of Antioxidants

#### 3.5.1. Superoxide Dismutase (SOD1)

**Figure 5:**
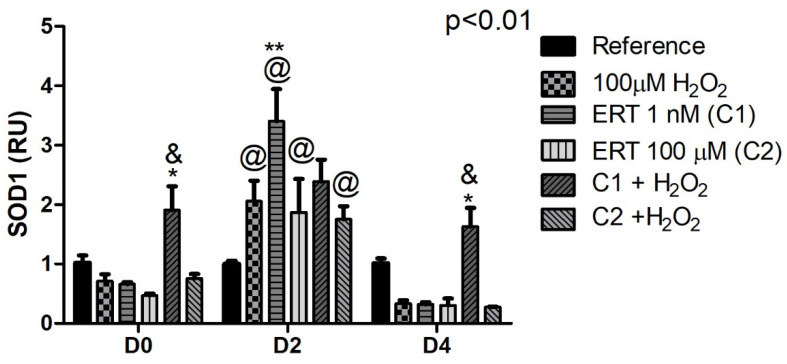
Relative expression of Superoxide Dismutase 1 (SOD1) in erythroid cells K562. Reference: K562 cells without H_2_O_2_ and not treated with ERT; 100 µM H_2_O_2_: Cells under stress induction with hydrogen peroxide; C1: cells translated with 1 nM ergothioneine (ERT); C2: cells translated with 100 µM ERT; C1 + 100 µM H_2_O_2_ and C2 + 100 µM H_2_O_2_: sets of cells treated with the same concentrations of ERT associated with stress induction; D0: before the differentiation process; D2: the beginning of cell differentiation; D4: end of the process; and RU: Relative Unit-fold-change of the transcripts related to the Reference subgroup in D0. The symbols indicate the following statistical differences: * Effect of treatment C1 + 100 µM H_2_O_2_ within each period, compared to treatment with peroxide (100 µM H_2_O_2_); ** Effect of treatment C1 compared to the reference; & Effect of lower concentration of ERT (C1) compared to higher (C2), both associated with the induction of oxidative stress (100 µM H_2_O_2_); and @ Effect of the differentiation period within each treatment, compared to its counterpart in D0 and D4. The representative values of the three pseudoreplicates were expressed as mean with standard error (±SEM). General Linear Models (GLM) with two-way ANOVA format, complemented by the Bonferroni test.

The comparisons of the relative gene expression of *SOD1* (Figure 5) showed an increase (up to ~3.5-fold) expression in the Peroxide, ERT C1, ERT C2, and ERT C2 + 100 µM H_2_O_2_ treatments (D2), while in the other treatments (Reference and ERT C1 + 100 µM H_2_O_2_) gene expression remained stable regardless of the differentiation period. Additionally, a combined effect of C1 + 100 µM H_2_O_2_ can be perceived when compared to the treatments Peroxide and ERT C2 + 100 µM H_2_O_2_.

#### 3.5.2. Catalase (CAT)

**Figure 6:**
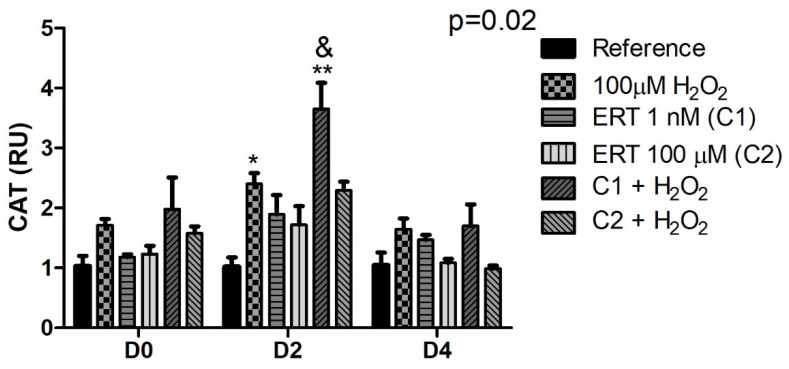
Relative expression of the catalase gene (*CAT*) in K562 erythroid cells. Reference: K562 cells without H_2_O_2_ and not treated with ERT; 100 µM H_2_O_2_: Cells under stress induction with hydrogen peroxide; C1: cells translated with 1 nM ergothioneine (ERT); C2: cells translated with 100 µM ERT; C1 + 100 µM H_2_O_2_ and C2 + 100 µM H_2_O_2_: sets of cells treated with the same concentrations of ERT associated with stress induction; D0: before the differentiation process; D2: the beginning of cell differentiation; D4: end of the process; and RU: Relative Unit-fold-change of the transcripts related to the Reference subgroup in D0. The symbols indicate the following statistical differences: * Effect of the Peroxide treatment, on day 2, compared to the reference; ** Effect of C1 + 100 µM H_2_O_2_ treatment within each period, compared to reference; and & Effect of lower concentration of ERT (C1) compared to higher (C2), both associated with H_2_O_2_. The representative values of the three pseudoreplicates were expressed as mean with standard error (±SEM). General Linear Models (GLM) with two-way ANOVA format, complemented by the Bonferroni test.

The comparisons of the relative gene expression of mRNA *CAT* (Figure 6) showed, on day 2, a ~2.5-fold increase of the Peroxide treatment (compared to the reference) and a ~3.5-fold increase in expression in the ERT C1 + 100 µM H_2_O_2_ treatment, in comparison with the treatments Reference (pattern also observed in D0) and ERT C2 + 100 µM H_2_O_2_, demonstrating an effect of ERT dependent on the addition of peroxide (100 µM H_2_O_2_).

#### 3.5.3. Glutathione Peroxidase 1 (GPX1)

**Figure 7:**
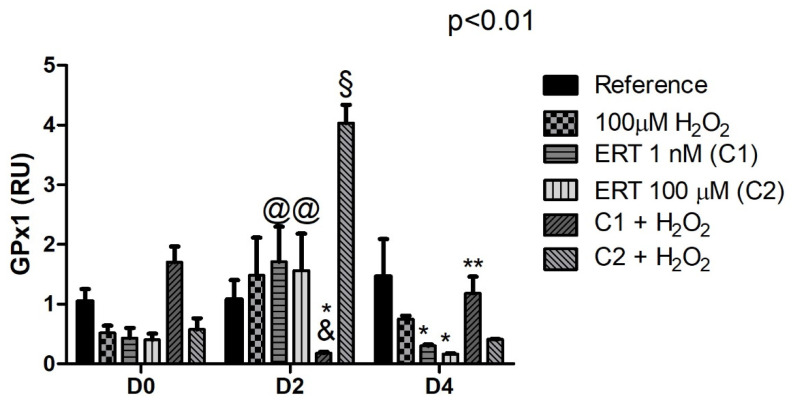
Relative expression of the Glutathione Peroxidase 1 (GPX1) gene in K562 erythroid cells. Reference: K562 cells without H_2_O_2_ and not treated with ERT; 100 µM H_2_O_2_: Cells under stress induction with hydrogen peroxide; C1: cells translated with 1 nM ergothioneine (ERT); C2: cells translated with 100 µM ERT; C1 + 100 µM H_2_O_2_ and C2 + 100 µM H_2_O_2_: sets of cells treated with the same concentrations of ERT associated with stress induction; D0: before the differentiation process; D2: the beginning of cell differentiation; D4: end of the process; RU: Relative Unit-fold-change of the transcripts related to the Reference subgroup in D0. The symbols indicate the following statistical differences: @ Effect of the differentiation period within each treatment, compared to its counterpart in D0 and D4; * Treatment effect, within each period, compared Peroxide treatment (100 µM H_2_O_2_); ** Effect of lower concentration of ERT (C1), on day 4, compared to higher concentration (C2), both associated with H_2_O_2_; & Decrease in gene expression resulting from the effect of the lower concentration of ERT (C1) compared to the higher (C2), both associated with the induction of oxidative stress (100 µM H_2_O_2_); and § Increase in gene expression resulting from the effect of a higher concentration of ERT (C2) compared to a lower one (C1), both associated with H_2_O_2_. The representative values of the three pseudoreplicates were expressed as mean with standard error (± SEM). General Linear Models (GLM) with two-way ANOVA format, complemented by the Bonferroni test.

Treatments ERT C1 and C2 showed a ~1.5-fold increase in the expression of *GPX1* at the beginning of erythroid differentiation (D2). Furthermore, changes of this transcript in a dose-dependent manner were observed, with C1 + H_2_O_2_ drastically inhibiting the expression (~8-fold decreased) while C2 + H_2_O_2_ exhibited a substantial increase (4-fold). However, the opposite is observed, more subtly, on day 4. Although not with statistical significance, D0 presents the same pattern (Figure 7).

#### 3.5.4. Thioredoxin (TRX)

**Figure 8:**
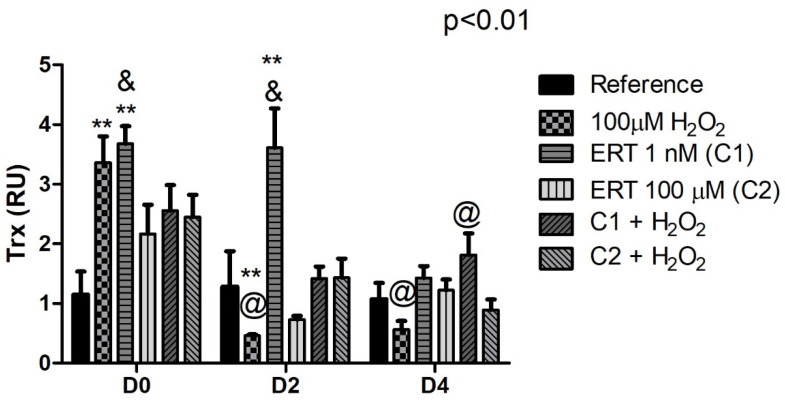
Relative expression of the thioredoxin (*TRX*) gene in K562 erythroid cells. Reference: K562 cells without H_2_O_2_ and not treated with ERT; 100 µM H_2_O_2_: Cells under stress induction with hydrogen peroxide; C1: cells translated with 1 nM ergothioneine (ERT); C2: cells translated with 100 µM ERT; C1 + 100 µM H_2_O_2_ and C2 + 100 µM H_2_O_2_: sets of cells treated with the same concentrations of ERT associated with stress induction; D0: before the differentiation process; D2: the beginning of cell differentiation; D4: end of the process; and RU: Relative Unit-fold-change of the transcripts related to the Reference subgroup in D0. The symbols indicate the following statistical differences: ** Effect of treatment within each period, compared to reference; & Effect of the lowest concentration of ERT (C1) compared to the highest (C2), within each period; and @ Effect of the differentiation period within each treatment, compared to its counterpart in D0. The representative values of the three pseudoreplicates were expressed as mean with standard error (±SEM). General Linear Models (GLM) with two-way ANOVA format, complemented by the Bonferroni test.

Peroxide treatment showed a ~3- and a 2-fold decrease in expression of *TRX* (days 2 and 4, respectively), while ERT C1 treatment exhibited an increased expression (~3.5-fold on days 0 and 2). Although not significant, a pattern of reduction prevention (day 2) can be observed when analyzing the relative expression of the treatments C1 + 100 µM H_2_O_2_ and C2 + 100 µM H_2_O_2_ (Figure 8).

#### 3.5.5. Peroxiredoxins (PRDXs)

**Figure 9:**
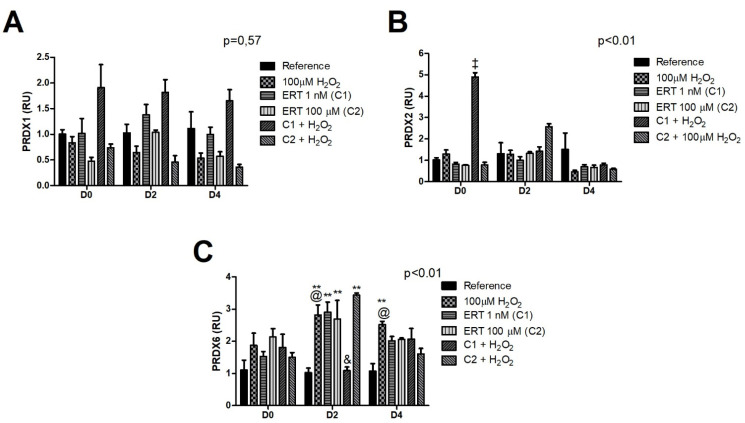
Relative expression of the Peroxiredoxins in K562 erythroid cells. (**A**)—Peroxiredoxin 1 (*PRDX1*). (**B**)—Peroxiredoxin 2 (*PRDX2*). (**C**)—Peroxiredoxin 6 (*PRDX6*). Reference: K562 cells without H_2_O_2_ and not treated with ERT; 100 µM H_2_O_2_: Cells under stress induction with hydrogen peroxide; C1: cells translated with 1 nM ergothioneine (ERT); C2: cells translated with 100 µM ERT; C1 + 100 µM H_2_O_2_ and C2 + 100 µM H_2_O_2_: sets of cells treated with the same concentrations of ERT associated with stress induction; D0: before the differentiation process; D2: the beginning of cell differentiation; D4: end of the process; and RU: Relative Unit-fold-change of the transcripts related to the Reference subgroup in D0. The symbols indicate the following statistical difference: ‡ Effect of ERT C1 + 100 µM H_2_O_2_ treatment compared to all treatments; ** Effect of treatment within each period, compared to reference; & Effect of lower concentration of ERT (C1) compared to higher (C2), both associated with the induction of oxidative stress (100 µM H_2_O_2_); and @ Effect of the differentiation period within each treatment, compared to its counterpart in D0. The representative values of the three pseudoreplicates were expressed as mean with standard error (±SEM). General Linear Models (GLM) with two-way ANOVA format.

The comparisons of the relative gene expression of the transcript *PRDX1* (Figure 9A) did not show statistically significant variations between treatments or periods of differentiation. Despite this, an antagonistic expression pattern can be observed according to the concentration of ERT administered in association with peroxide, in which C1 induces, and C2 inhibits *PRDX1* expression, especially on days 2 and 4. *PRDX2* (Figure 9B) showed a constant expression in all treatments during the observed periods, except for ERT C1 + 100 µM H_2_O_2_ treatment (day 0), in which there is a ~5-fold increase in gene expression. Although not significant, the same pattern can be observed, on day 2, for the ERT C2 + 100 µM H_2_O_2_ treatment. On day 2 of *PRDX6* gene expression (Figure 9C), an increased level of transcripts (up to ~3.5-fold) in the treatments Peroxide, ERT C1, ERT C2, and ERT C2 + 100 µM H_2_O_2_ was observed. Furthermore, in the same period, a decreased expression in ERT C1 can be observed, with the physiological concentration being restored.

### 3.6. Proteasome

**Figure 10:**
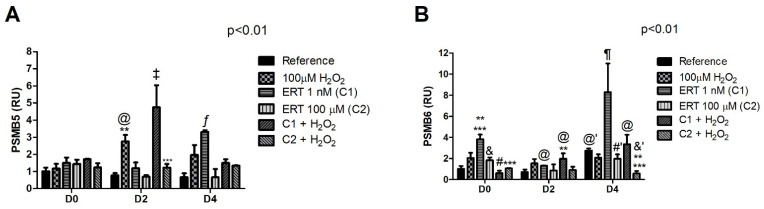
Relative expression of the Proteasome (*PSMB5* and *PSMB6*) in K562 erythroid cells. (**A**)—Proteasome 20S Subunit Beta 5 (*PSMB5*). (**B**)—Proteasome 20S Subunit Beta 1 or Proteasome Subunit Beta Type 6 (*PSMB6*). Reference: K562 cells without H_2_O_2_ and not treated with ERT; 100 µM H_2_O_2_: Cells under stress induction with hydrogen peroxide; C1: cells translated with 1 nM ergothioneine (ERT); C2: cells translated with 100 µM ERT; C1 + 100 µM H_2_O_2_ and C2 + 100 µM H_2_O_2_: sets of cells treated with the same concentrations of ERT associated with stress induction; D0: before the differentiation process; D2: the beginning of cell differentiation; D4: end of the process; and RU: Relative Unit-fold-change of the transcripts related to the Reference subgroup in D0. The symbols indicate the following statistical differences: @ Effect of the differentiation period within each treatment, compared to its counterpart in D0; @’ Effect of the differentiation period within each treatment, compared to its counterpart in D0 and D2; ** Effect of treatment within each period, compared to treatment Reference; *** Effect of treatment within each period, compared to treatment 100 µM H_2_O_2_; ‡ Effect of ERT C1 + 100 µM H_2_O_2_ treatment compared to all treatments; ¶ Increased levels of transcripts in the ERT C1 group compared to all treatments; ƒ Effect of ERT C1 group compared to the others (except for the 100 µM H_2_O_2_ group in D2-non-significant difference); # Effect of lowest concentration of ERT (C1) compared to ERT C1 + 100 µM H_2_O_2_; #’ Effect of the highest concentration of ERT (C2) compared to the ERT C2 + 100 µM H_2_O_2_; & Effect of the highest concentration of ERT (C2) compared to the lowest (C1), within each period; and &’ Effect of the highest concentration of ERT (C2) compared to the lowest (C1), both associated with 100 µM H_2_O_2_. The representative values of the three pseudoreplicates were expressed as mean with standard error (±SEM). General Linear Models (GLM) with two-way ANOVA format, complemented by the Bonferroni test.

The comparisons of the relative gene expression of *PSMB5* (Figure 10A) showed a constant expression in all treatments during D0. Although, during the beginning of the erythroid differentiation process (D2), a ~2.5- and 4.5-fold increased expression can be observed in the Peroxide and ERT C1 + 100 µM H_2_O_2_ treatments, respectively. However, at the end of the differentiation process (D4), the expression returns to a constant state in all treatments associated with H_2_O_2_, even though a seemed elevated expression can be observed in the Peroxide treatment. All treatments with ERT associated with the oxidation induction, regardless of the period of differentiation, present expression levels equivalent to the physiological concentration, except for the ERT C1 + 100 µM H_2_O_2_ treatment in D2. Regarding *PSMB6* expression (Figure 10B), on D0 and D4, a decreased pattern of expression can be seen in the treatments ERT C1 + 100 µM H_2_O_2_ and C2 + 100 µM H_2_O_2_ when compared with the Peroxide, ERT C1, and C2, respectively. Lastly, on the D4, higher transcripts levels of both *PSMB5* and *6* (~3- and 8-fold increase, respectively) in the treatment ERT C1 can be observed.

### 3.7. Overview of the Expression Pattern of the Genes Involved in the Redox Adaptation Mechanisms in K562 Cells

**Figure 11:**
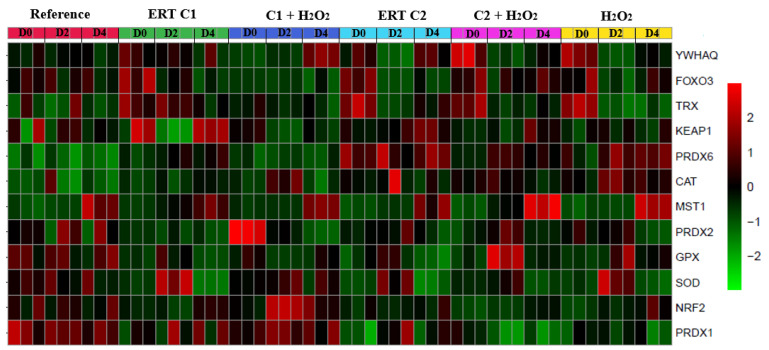
Overview of the expression pattern of the genes involved in the redox adaptation mechanisms in K562 cells. Genes are clustered hierarchically (full clustering method) using Euclidean correlation as the distance metric, with colors ranging from green (lowest) to red (highest), indicating the level of gene expression in each treatment and period evaluated. Reference: K562 cells without H_2_O_2_ and not treated with ERT; 100 µM H_2_O_2_: Cells under stress induction with hydrogen peroxide; C1: cells translated with 1 nM ergothioneine (ERT); C2: cells translated with 100 µM ERT; C1 + 100 µM H_2_O_2_ and C2 + 100 µM H_2_O_2_: sets of cells treated with the same concentrations of ERT associated with stress induction; D0: before the differentiation process; D2: the beginning of cell differentiation; and D4: end of the process.

We created a Heat map to improve the overview visualization of our data set. Thus, Figure 11 summarizes the transcription level changes observed in our work, with colors ranging from green (lowest) to red (highest), indicating the level of gene expression in each treatment and period evaluated.

### 3.8. Associations between Expressions of Redox Pathways Genes and Antioxidants in Erythroid Cells

The GRM analysis provides the degree of association between a set of interrelated independent variables and a dependent variable through multiple associations (R). In other words, the coefficient R indicates the contribution of the group indicated as independent in the increase or decrease in the levels of the dependent variable. Among the results of GRM, the importance of the redox pathways analyzed in the production of antioxidant enzymes and proteasomal genes (except for *PRDX6* and *PSMB6*, whose correlations were non-significant). Consequently, the maintenance of cell integrity and redox homeostasis in K562 erythroid cells stands out. Additionally, it is important to highlight that the expression of γ-globin was not correlated with any of the analyzed genes. The results obtained in this analysis can be seen in Table 1.

Furthermore, the GRM analysis also provides the partial correlation (r), i.e., the individual contribution of each independent variable on the increasing or decreasing of the dependent variable, after the control for all other independent variables in the equation (Table 2). Regarding the expression of *PRDX1*, *SOD1*, *CAT*, and *PSMB5*, the Keap1/Nrf2/ARE pathway showed a greater contribution or involvement in the production of mRNA of these genes. On the other hand, the FoxO3-MST1 pathway seems to be associated with the production of *PRDX2* and *TRX* enzymes in K562 erythroid cells.

## 4. Discussion

The unique properties of ERT—a naturally occurring amino acid—since its discovery, have puzzled researchers for more than a century [24]. Although the ERT’s physiological role is not yet well established, there is increasing evidence of its involvement in the homeostasis of erythroid cells. Studies in erythroid models have shown that this antioxidant is abundantly accumulated in hematopoietic cells [44], being even the second most abundant thiol in mature erythrocytes [45], whose reasons for this abundance in hematopoietic tissues are still unknown. Additionally, a study performed by Kupers et al. suggests that ERT is selectively depleted when compared to GSH in sickle cell erythrocytes, suggesting a specialized function in the protection of red blood cells [46], especially sickle cells, since they have levels twice decreased of this antioxidant [40].

Furthermore, studies have suggested that ERT is preferentially accumulated in tissues predisposed to oxidative stress and inflammation and may even be concentrated in tissue injury sites by cellular modulation of ETT levels [25,47,48,49]. These unique ERT capabilities, reported in many cells, animal models, and even population studies, suggest that ERT cytoprotective skills can be useful against numerous human disorders [50]. Thus, to the authors’ knowledge, the present study produced a unique opportunity to understand possible mechanisms of ERT action on proliferation, differentiation, and redox adaptation processes in K562 erythroid cells to provide subsidies for its use as an adjuvant in the treatment of hematological diseases presenting oxidative stress as a pathophysiological consequence.

High cell viability was maintained during all experimental days, regardless of adding the protective agent (ERT) and the stressor (H_2_O_2_). However, in vivo studies [51] have shown that in healthy cells, the concentration of H_2_O_2_ rarely exceeds 1–15 µM, being even lower in erythrocytes, as reported by Benfeitas et al., in which H_2_O_2_ concentrations are in the nM range [52]. Thus, even though there were no changes in viability, it is plausible to assume that an increase in the oxidative load (due to the addition of H_2_O_2_) could shift the organism to a low-intensity oxidative stress state. This subtype of oxidative stress is characterized by the oxidation of the most reactive cellular components and induction of the redox-dependent response [53].

This cellular response is associated with the varying intracellular concentrations of H_2_O_2_ throughout the cell [54], which provides a theoretical basis for understanding the H_2_O_2_ signaling and redox relays [55]. Nonetheless, it is worth mentioning that the production of reactive oxygen species was not measured by fluorescent probe following oxidative treatment.

Furthermore, the low mortality rate observed in the treatment with the stressor agent can be explained by the endogenous production of antioxidant agents during the differentiation process induced by hemin [56]. This production is associated with the set of morphological and physiological changes triggered by the administration of hemin and hydroxyurea that increases the complexity of this cell type [57,58,59,60].

A known regulator of crucial activities for the maintenance of erythroid cells is FoxO3. This transcription factor is considered an essential factor for the maintenance of cellular homeostasis during the hematopoiesis process. FoxO3 signaling networks, their regulators, and coactivators in hematopoietic stem cells and erythroid progenitors act in order to integrate and transmit multiple signals that cooperate in regulating the erythroid cell gene expression program [61,62]. Among the activities carried out by this transcription factor are proliferation, differentiation, γ-globin expression, and transcriptional regulation in terminal erythroblastic maturation [62,63,64,65,66]. Although, in this study, no correlation was seen between γ-globin and the expression of FoxO3 (or Nrf2–a known HbF stimulator), in disagreement with the literature [66,67]. However, an increase (up to ~6.5-fold) in the γ-globin mRNA levels can be associated with ERT treatments, thus suggesting its association with HbF expression in K562 cells. Along these lines, it is proposed that ERT treatment would be especially beneficial for hemoglobinopathies, such as sickle cell disease (SCD), a group of inherited diseases whose therapeutic options currently available are extremely limited. The increased expression of HbF, a known modulator of SCD, is associated with decreased clinical manifestations [68].

In this context, by a mechanism not yet elucidated, the study performed by Nakamura et al. (2007) suggests a role of ERT in the differentiation, maturation, and growth processes of erythroid cells since the absence of this thiol culminated in the reduction of these processes in K562 cells [69]. The present study corroborates this hypothesis, suggesting, for the first time in the literature, a regulatory axis for erythroid differentiation in K562 cells, in which the low concentration of ERT acts indirectly, on erythroid proliferation, through the induction of *FOXO3* expression associated with the induction of one of its target genes, *TRX*, evidenced by a ~3.5-fold increase of their mRNA levels, on D0 and 2 of ERT C1 treatment.

In addition to modulating different cellular processes, including cell proliferation [70], Trx is one of the primary cellular redox buffers whose relative expression is provenly associated with *FOXO3*, as demonstrated by the multiple association analysis. To this author’s knowledge, such association was only reported in bovine aorta endothelial cells [71]. From these results, there is significant evidence for the establishment of the ERT-FoxO3-Trx axis as a potential therapeutic target by modulating the expression of *FOXO3*, a positive regulator not only of erythroid differentiation but also essential for the maintenance of redox homeostasis, through controlling Trx bioavailability, and consequentially to PRDX reduction (except for PRDX6) [72,73], which reinforces the therapeutic benefits of ERT.

The influence of ERT on the modulation of Nrf2 has already been reported by Hseu et al. (2015) [31]. We also observed an indirect action, demonstrating an interesting cytoprotective role against the oxidative environment generated by adding peroxide. K562 cell treatment, ERT C1 + 100μM H_2_O_2_, showed increased levels of *NRF2*, *PRDX1*, *SOD1*, and *CAT* transcripts on all evaluated days and *PRDX2* on day 0. The association of this transcription factor with the expression pattern of these antioxidants was confirmed through the GRM analysis. On the other hand, in K562 cells treated with ERT C2 + 100 μM H_2_O_2_, activation of the redox protection pathway MST1-FoxO3 can be seen. The *FOXO3* expression seems to be related to the induction of *PRDX2*, *PRDX6*, and *GPX1*, on day 2, suggesting an indirect action of ERT in the combat of H_2_O_2_ by stimulating the expression of the *FOXO3* and *MST1* associated with a decreased expression of *YWHAQ* (14-3-3 chaperone).

Considering these results, we proposed a regulatory mechanism in which ERT influences the expression of antioxidant genes and, consequently, the antioxidant response of erythroid cells incubated with 100 µM H_2_O_2_. It is worthy of mention that some limitations of the present study need to be acknowledged, such as the evaluation of post-translational modifications of Nrf2 and FoxO3, as well the protein levels of the biomarkers. Nevertheless, this work is an outstanding hypothesis-generating study, assuming similar patterns to the transcripts expressed at the protein level. In addition, the proposed regulation model suggests a dynamic relationship exists between the redox signaling pathways of the investigated transcription factors triggered as part of an adaptive response to the addition of H_2_O_2_.

This propositioned regulatory mechanism involves multiple steps, starting with the cell signaling generated by the added peroxide itself. H_2_O_2_ can lead to the reversible oxidation of redox-sensitive Cys residues in the active sites of the phosphatase and tensin homologue (PTEN), a known AKT negative regulator, resulting in the negative modulation of FoxO3 (Figure S2–supplementary material) [74,75,76,77]. ETT transports the ERT with very high efficiency (50–200 μL/min/mg of protein; *K*_m_ value of about 20 μM) into cells [32]. Additionally, its uptake increases linearly in K562 cells. These facts allow the assumption that both ERT concentrations administered are internalized minutes after their administration [69]. It is assumed that in the low concentration (ERT C1 + 100 µM H_2_O_2_ treatment), the ERT cannot significantly reduce the H_2_O_2_ intracellular signaling. Thus, maintaining FoxO3 in the nucleus. In the ERT C1 + 100 µM H_2_O_2_ treatment, a cytoplasmatic FoxO3 concentration is proposed (first regulatory level). The modulation of the FoxO3 allows the proposition that a second transcription factor is activated, the Nrf2, whose expression was increased in this treatment. Guan et al. were the first to suggest a dynamic regulation between these transcription factors by describing a negative regulation of Keap1 caused by FoxO3 depletion [78], a situation that could be occurring here, with our results showing an increase in *NRF2* expression (D2 of the ERT C1 + 100 µM H_2_O_2_ treatment) and, consequently, suggesting an activation of the Nrf2-ARE pathway.

According to Dare et al. (2021), ERT can be intimately related to the activation of the Nrf2-ARE pathway. The authors showed, in silico, that ERT could directly bind to Nrf2 by substituting the IVV-ligand with ERT in the Nrf2-IVV crystal complex, leading to Nrf2-Keap1 complex dissociation, nuclear accumulation of the freed Nrf2, thus the activation of antioxidant genes [79]. Moreover, indirectly, ERT could activate the Nrf2 pathway through the oxidized intermediates of the ERT (i.e., sulfonated [ESO_3_H] and desulfurized [EH] forms, also known as hercinin and disulfide [ESSE]) generated from the direct detoxification of H_2_O_2_ [80]. These intermediates could destabilize the Keap1-Cul3-Rbx1 complex (e.g., through oxidation of Keap1 cysteines), stimulating the nuclear accumulation of Nrf2 (Figure S3–supplementary material). Regardless of the action of the proposed mechanisms, the greater expression of *NRF2* suggests a probable nuclear accumulation of this TF, where it will act as a positive regulator of antioxidants genes in the ERT C1 + 100 µM H_2_O_2_ treatment (second level of the proposed regulatory mechanism). Thus, an ERT’s dose-dependent response is observed via the activated redox pathway selected to combat oxidative stress.

Due to its role as an oxidative stress sensor and in cell signaling, the PRDX1–whose expression increased in this treatment (data corroborated by GRM), leads to the proposition of the last step for the mechanism. As described by Hopkins et al., PRDX1 can interact with FoxO3 in an oxidative stress-dependent manner influencing its subcellular location [81]. Furthermore, PRDX1 can also propagate peroxide-mediated signals in a spatially confined location, generating H_2_O_2_ gradients around sites where signaling proteins are concentrated [10,82]. These characteristics allow H_2_O_2_ to accumulate at substantial levels under certain circumstances, thus facilitating H_2_O_2_-dependent signaling [82,83]. Moreover, a slower rate of reactivation of hyperoxidized PRDXs (via sulfiredoxin-dependent reduction) is proposed to be a built-in mechanism, which allows sufficient time for H_2_O_2_ accumulation and signal propagation [84]. Thus, in the treatment ERT C1+ 100 µM H_2_O_2_, it is assumed that PRDX1 acts reinforce FoxO3′s cytoplasmic concentration. This retention could be associated with recycling or degradation (levels of *PSMB5* in this treatment support the latter).

**Figure 12:**
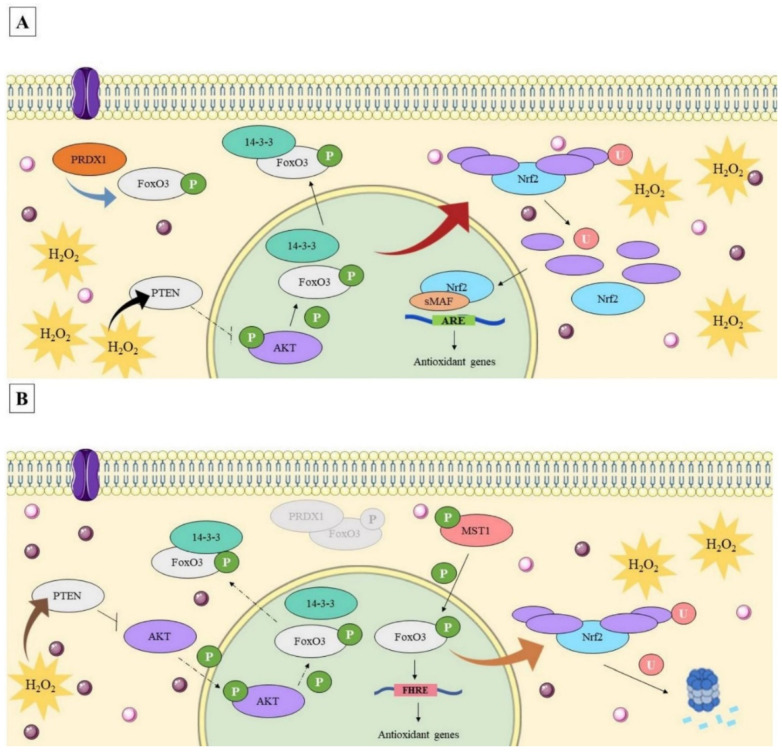
Proposed mechanism of action for cytoprotective action of ergothioneine in erythroid K562 cells under oxidative stress. (**A**)—Proposed mechanism for the low concentration of ergothioneine. Ergothioneine (ERT), represented by a circle (purple, reduced form and pink represents the ERT oxidized intermediaries), is internalized by its specific transporter ETT (shown in purple, on the left side of the figure) acts directly detoxifying the hydrogen peroxide (H_2_O_2_). However, the H_2_O_2_ resulting concentration is enough to activate the PI3K/AKT signaling pathway (thick black arrow), which results in the cytoplasmic concentration of FoxO3 (thru the chaperone 14-3-3). The reduced expression of *FOXO3* results in the expression of a second transcription factor, *NRF2* (red arrow) which, once activated, acts on the transcription of antioxidant enzymes (via Nrf2-ARE pathway), such as *PRDX1*. This oxidative stress sensor acts in the last stage of this regulation (blue arrow), aiding in the cytoplasmic maintenance of FoxO3. (**B**)—Proposed mechanism for the high concentration of ergothioneine. ERT acts directly detoxifying the H_2_O_2_ and, in this concentration, ERT detoxifies enough H_2_O_2_ to prevent the activation of the PI3K/AKT pathway and, consequently, the translocation of the FoxO3 to the cytoplasm (thick brown arrow). The high nuclear concentration of FoxO3 and the increase in its expression, especially on D2, results in a decreased expression of *NRF2* (orange arrow) which is forwarded for degradation via proteasome. *PRDX1* present a low expression in this treatment. Thus, PRDX1 is not enough to concentrate FoxO3 in the cytoplasm (represent in the center as gray proteins) buttressing the proposed theory of the transactivation of the target genes via FoxO3 pathway. ARE: Human Antioxidant Response Element; ERT: ergothioneine; FHRE: FoxO responsive element; FoxO3, Forkhead box O3 protein; Nrf2, erythroid nuclear factor 2 related to factor 2; P: phosphorylation; PER: hydrogen peroxide; PTEN: Phosphatase and tensin homology; sMAF, small Maf protein; U, ubiquitination. Triggered line: inactive pathway; Continuous line: active pathway. For interpretation of the references to color in this figure legend, the reader is referred to the web version of this article. Source: This figure was created by the authors adapting images from Servier Medical Art Commons Attribution 3.0 Unported License ((http://smart.servier.com), accessed on 15 January 2021).

As previously stated, in the C2 + H_2_O_2_ treatment, the ERT concentration should significantly reduce the H_2_O_2_-signaling, leading to the activation of the PI3K/AKT pathway and FoxO3 nuclear retention. Accordingly, an increase in *FOXO3* transcripts is observed, especially on D2. Additionally, a decreased expression of *NRF2* is observed, possibly due to the dynamic relationship between FoxO3-Nrf2 previously mentioned. Therefore, by indirectly decreasing H_2_O_2_-signaling, ERT modulates the FoxO3 signaling pathway and its target genes. Additionally, *PRDX1* low transcription levels reinforce this proposed theory [20,85]. The regulatory mechanisms are summarized in Figure 12.

Grune, Reinheckel, and Davies (1996) were the first to demonstrate that mild oxidative stress can increase the intracellular degradation of both short-lived and long-lived proteins in K562 cells. They proposed that the oxidative modification of substrate proteins is the major cause of increased cellular protein degradation following oxidative stress [86]. The observed proteasomal genes expression (*PSMB5* and *6*) allowed for the proposal of an association between ERT and proteasome in a period and dose-dependent manner. To the author’s knowledge, this association is the first to ever be made in human cells. The only mention between ERT and proteasome so far was made by Takeda et al. (2010) in a study involving the fission yeast, *Schizosaccharomyces pombe*, in which was observed accumulation of ERT in the proteasome mutants *MTS3-1* and *PTS1-727* [87].

The obtained data suggested that the high concentration of ERT is responsible for the maintenance of the physiological concentration or, in some cases, led to a reduction of the transcription rate of the analyzed proteasomal subunits (especially for *PSMB6* during oxidative stress). This decline could be associated with the direct depletion of H_2_O_2_ by ERT, which could lead to a lower concentration of oxidized proteins and, consequently, lessen the need for new proteasome synthesis. An exception to this pattern is seen in ERT C1 + 100 µM H_2_O_2_ treatment (D2), whose increased expression (of ~4.5-fold for *PSMB5* and 2-fold for *PSMB6*, respectively) could be associated with the Keap1/Nrf2/ARE pathway activation, since *PSMB5* and *6* are well-known targets genes of this transcript factor [17,18] or to the proposed degradation of FoxO3. Lastly, an interesting case is observed in the D4, where high transcripts levels of both *PSMB5* and *6* in ERT C1 treatment can be observed (~3- and 8-fold increase, respectively). The elevated transcript levels of these proteasome subunits suggest a molecular moment of increased protein degradation. Due to the lack of oxidative damage in this treatment (no addition of H_2_O_2_), this increase could be associated with the primary proteolytic route for short-lived, misfolded, and damaged proteins [88].

## 5. Conclusions

The administration of ERT in erythroid cells K562 showed period and dose-dependent effects against oxidative stress induced by H_2_O_2_, with direct actions on the detoxification of the stressor and indirect on the levels of the evaluated transcripts. These observations enabled the proposition of a regulatory mechanism among FoxO3-Keap1-Nrf2 with a fundamental role in redox homeostasis in the biological model studied, in which both Nrf2-ARE and MST1-FoxO3 are involved in redox homeostasis. In contrast, the ERT-FoxO3-Trx pathway is involved in erythroid proliferation and differentiation. Lastly, ERT is proposed to be also associated with γ-globin expression. It is worth noting that future studies of protein expression and subcellular immunostaining of the analyzed pathways are necessary to prove the proposed crosstalk hypothesis between FoxO3 and Nrf2 for erythroid cells. Finally, it is suggested that ERT should be considered a key element in future investigations of therapeutic alternatives for treating hematological diseases.

## Figures and Tables

**Table 1:** Multiple associations between the relative expressions of members of redox pathways essential for antioxidant defense in erythroid cells and their respective antioxidant enzymes.

Dependent Variable	Independent Variable
	Members of FOXO3 and Nrf2 pathways
*PRDX1*	**R = 0.81; *p* < 0.00**
*PRDX2*	**R = 0.77; *p* < 0.00**
*PRDX6*	R = 0.35; *p* = 0.24
*SOD1*	**R = 0.46; *p* = 0.03**
*CAT*	**R = 0.66; *p* < 0.00**
*GPX1*	**R = 0.45; *p* = 0.04**
*TRX*	**R = 0.75; *p* < 0.00**
*PSMB5*	**R = 0.50; *p* = 0.01**
*PSMB6*	R = 0.38; *p* = 0.15
*γ-globin*	R = 0.29; *p* = 0.47

R: multiple association coefficient (General Regression Model analysis, multiple regression design); *p* < 0.05 was considered to be statistically significant (in bold).

**Table 2:** Individual associations between members of the redox signaling pathways and the antioxidant and proteasomal genes analyzed.

Dependent Variable	Independent Variable				
	*Nrf2*	*Keap1*	*FOXO3*	*MST1*	*14-3-3*
*PRDX1*	**r = 0.72**; ***p* < 0.00**	**r = 0.49**; ***p* < 0.00**	r = 0.23; *p* = 0.10	r = −0.25; *p* = 0.06	**r = 0.18**; ***p* = 0.03**
*PRDX2*	**r = 0.28**; ***p* = 0.04**	**r = 0.69**; ***p* < 0.00**	**r = 0.29**; ***p* = 0.03**	r = 0.07; *p* = 0.58	**r = −0.25**; ***p* < 0.00**
*SOD1*	**r = 0.31**; ***p* = 0.02**	r = 0.15; *p* = 0.27	r = 0.11; *p* = 0.41	**r = −0.32**; ***p* = 0.01**	r = 0.08; *p* = 0.49
*CAT*	**r = 0.63**; ***p* < 0.00**	r = 0.10; *p* = 0.45	r = 0.08; *p* = 0.55	r = −0.19; *p* = 0.18	r = −0.07; *p* = 0.46
*GPX1*	r = −0.24; *p* = 0.08	**r = 0.33**; ***p* = 0.01**	r = 0.09; *p* = 0.49	r = −0.20; *p* = 0.15	r = −0.10; *p* = 0.41
*TRX*	r = 0.04; *p* = 0.74	r = 0.26; *p* = 0.06	**r = 0.45**; ***p* < 0.00**	r = −0.04; *p* = 0.76	**r = 0.49**; ***p* < 0.00**
*PSMB5*	r = −0.10; *p* = 0.44	**r = 0.50**; ***p* < 0.00**	r = −0.00; *p* = 0.95	r = −0.13; *p* = 0.29	r = 0.07; *p* = 0.58

r: partial correlation coefficient that demonstrates the individual effect of each independent variable tested. *p* < 0.05 was considered to be statistically significant (in bold).

## Data Availability

The data that support the findings of this study are available from the corresponding author, D.S., upon reasonable request.

## Supplementary Materials

The following supporting information can be downloaded at: https://www.mdpi.com/article/10.3390/genes13122368/s1, Table S1: Primers sequences; Figure S1: Viability of K562 differentiated cells under the different experimental conditions tested; Figure S2: Cell signaling generated by hydrogen peroxide and its modulatory effect on the FoxO3 transcription factor; Figure S3: Proposed mechanisms of action for activating the Nrf2-ARE pathway in the treatment with the lowest concentration of ergothioneine in erythroid cells K562 under oxidative stress.

## References

[1] Jones D.P., Sies H. (2015). The Redox Code. Antioxid. Redox Signal..

[2] Poprac P., Jomova K., Simunkova M., Kollar V., Rhodes C.J., Valko M. (2017). Targeting Free Radicals in Oxidative Stress-Related Human Diseases. Trends Pharmacol. Sci..

[3] Liguori I., Russo G., Curcio F., Bulli G., Aran L., Della-Morte D., Gargiulo G., Testa G., Cacciatore F., Bonaduce D. (2018). Oxidative Stress, Aging, and Diseases. Clin. Interv. Aging.

[4] Vona R., Sposi N.M., Mattia L., Gambardella L., Straface E., Pietraforte D. (2021). Sickle Cell Disease: Role of Oxidative Stress and Antioxidant Therapy. Antioxidants.

[5] Singh A., Kukreti R., Saso L., Kukreti S. (2019). Oxidative Stress: A Key Modulator in Neurodegenerative Diseases. Molecules.

[6] Lu J., Wang Z., Cao J., Chen Y., Dong Y. (2018). A Novel and Compact Review on the Role of Oxidative Stress in Female Reproduction. Reprod. Biol. Endocrinol..

[7] Pala F.S., Gürkan H. (2008). The Role of Free Radicals in Ethiopathogenesis of Diseases. Adv. Mol. Biol..

[8] Sies H., Berndt C., Jones D.P. (2017). Oxidative Stress. Annu. Rev. Biochem..

[9] Halliwell B. (2013). The Antioxidant Paradox: Less Paradoxical Now?. Br. J. Clin. Pharmacol..

[10] Lei X.G., Zhu J.H., Cheng W.H., Bao Y., Ho Y.S., Reddi A.R., Holmgren A., Arnér E.S.J. (2015). Paradoxical Roles of Antioxidant Enzymes: Basic Mechanisms and Health Implications. Physiol. Rev..

[11] Poljsak B., Milisav I. (2012). The Neglected Significance of “Antioxidative Stress”. Oxid. Med. Cell. Longev..

[12] Cuadrado A., Manda G., Hassan A., Alcaraz M.J., Barbas C., Daiber A., Ghezzi P., León R., López M.G., Oliva B. (2018). Transcription Factor NRF2 as a Therapeutic Target for Chronic Diseases: A Systems Medicine Approach. Pharmacol. Rev..

[13] Tauber S., Steinbrenner H., Klotz L.-O. (2020). FoxO Transcription Factors in the Control of Redox Homeostasis and Fuel Metabolism.

[14] Chen X., Cao X., Xiao W., Li B., Xue Q. (2020). PRDX5 as a Novel Binding Partner in Nrf2-Mediated NSCLC Progression under Oxidative Stress. Aging.

[15] Ma Q. (2013). Role of Nrf2 in Oxidative Stress and Toxicity. Annu. Rev. Pharmacol. Toxicol..

[16] Macari E.R., Lowrey C.H. (2011). Induction of Human Fetal Hemoglobin via the NRF2 Antioxidant Response Signaling Pathway. Blood.

[17] Kwak M.-K., Wakabayashi N., Greenlaw J.L., Yamamoto M., Kensler T.W. (2003). Antioxidants Enhance Mammalian Proteasome Expression through the Keap1-Nrf2 Signaling Pathway. Mol. Cell. Biol..

[18] Kwak M.K., Wakabayashi N., Itoh K., Motohashi H., Yamamoto M., Kensler T.W. (2003). Modulation of Gene Expression by Cancer Chemopreventive Dithiolethiones through the Keap1-Nrf2 Pathway: Identification of Novel Gene Clusters for Cell Survival. J. Biol. Chem..

[19] Boccitto M., Kalb R. (2011). Regulation of Foxo-Dependent Transcription by Post-Translational Modifications. Curr. Drug Targets.

[20] Kodani N., Nakae J. (2020). Tissue-Specific Metabolic Regulation of FOXO-Binding Protein: FOXO Does Not Act Alone. Cells.

[21] Wang X., Hu S., Liu L. (2017). Phosphorylation and Acetylation Modifications of FOXO3a: Independently or Synergistically?. Oncol. Lett..

[22] Melville D.B. (1959). Ergothioneine. Vitam. Horm..

[23] Cheah I.K., Halliwell B. (2021). Ergothioneine, Recent Developments. Redox Biol..

[24] Cheah I.K., Halliwell B. (2012). Ergothioneine; Antioxidant Potential, Physiological Function and Role in Disease. Biochim. Biophys. Acta-Mol. Basis Dis..

[25] Halliwell B., Cheah I.K., Tang R.M.Y. (2018). Ergothioneine—A Diet-Derived Antioxidant with Therapeutic Potential. FEBS Lett..

[26] Cheah I.K., Tang R.M.Y., Yew T.S.Z., Lim K.H.C., Halliwell B. (2017). Administration of Pure Ergothioneine to Healthy Human Subjects: Uptake, Metabolism, and Effects on Biomarkers of Oxidative Damage and Inflammation. Antioxid. Redox Signal..

[27] Forster R., Spézia F., Papineau D., Sabadie C., Erdelmeier I., Moutet M., Yadan J.C. (2015). Reproductive Safety Evaluation of L-Ergothioneine. Food Chem. Toxicol..

[28] Marone P.A., Trampota J., Weisman S. (2016). A Safety Evaluation of a Nature-Identical l-Ergothioneine in Sprague Dawley Rats. Int. J. Toxicol..

[29] Turck D., Bresson J., Burlingame B., Dean T., Fairweather-Tait S., Heinonen M., Hirsch-Ernst K.I., Mangelsdorf I., McArdle H.J., Naska A. (2017). Statement on the Safety of Synthetic L-ergothioneine as a Novel Food—Supplementary Dietary Exposure and Safety Assessment for Infants and Young Children, Pregnant and Breastfeeding Women. EFSA J..

[30] Song T.Y., Chen C.L., Liao J.W., Ou H.C., Tsai M.S. (2010). Ergothioneine Protects against Neuronal Injury Induced by Cisplatin Both in Vitro and in Vivo. Food Chem. Toxicol..

[31] Hseu Y.C., Lo H.W., Korivi M., Tsai Y.C., Tang M.J., Yang H.L. (2015). Dermato-Protective Properties of Ergothioneine through Induction of Nrf2/ARE-Mediated Antioxidant Genes in UVA-Irradiated Human Keratinocytes. Free Radic. Biol. Med..

[32] Gründemann D., Harlfinger S., Golz S., Geerts A., Lazar A., Berkels R., Jung N., Rubbert A., Schömig E. (2005). Discovery of the Ergothioneine Transporter. Proc. Natl. Acad. Sci. USA.

[33] Grigat S., Harlfinger S., Pal S., Striebinger R., Golz S., Geerts A., Lazar A., Schömig E., Gründemann D. (2007). Probing the Substrate Specificity of the Ergothioneine Transporter with Methimazole, Hercynine, and Organic Cations. Biochem. Pharmacol..

[34] Jelkmann W. (2013). Physiology and Pharmacology of Erythropoietin. Transfus. Med. Hemother..

[35] Paula C.P.D. (2020). Efeito da Melatonina na Proteção Contra Estresse Oxidativo em Células Eritrocitárias K562. Ph.D. Thesis.

[36] Rowley P.T., Ohlsson-Wilhelm B.M., Farley B.A., LaBella S. (1981). Inducers of Erythroid Differentiation in K562 Human Leukemia Cells. Exp. Hematol..

[37] Tennanth J. (1964). Evaluation of the Trypan Blue Technique for Determination of Cell Viability. Transplantation.

[38] da Silva D.G.H., Ricci O., de Almeida E.A., Bonini-Domingos C.R. (2015). Potential Utility of Melatonin as an Antioxidant Therapy in the Management of Sickle Cell Anemia. J. Pineal Res..

[39] Cheah I.K., Feng L., Tang R.M.Y., Lim K.H.C., Halliwell B. (2016). Ergothioneine Levels in an Elderly Population Decrease with Age and Incidence of Cognitive Decline; a Risk Factor for Neurodegeneration?. Biochem. Biophys. Res. Commun..

[40] Chaves N.A., Alegria T.G.P., Dantas L.S., Netto L.E.S., Miyamoto S., Bonini Domingos C.R., da Silva D.G.H. (2019). Impaired Antioxidant Capacity Causes a Disruption of Metabolic Homeostasis in Sickle Erythrocytes. Free Radic. Biol. Med..

[41] Livak K.J., Schmittgen T.D. (2001). Analysis of Relative Gene Expression Data Using Real-Time Quantitative PCR and the 2-ΔΔCT Method. Methods.

[42] Quinn G.P., Keough M.J. (2002). Experimental Design and Data Analysis for Biologists.

[43] McDonald J.H. (2014). Handbook of Biolological Statistics.

[44] Tschirka J., Kreisor M., Betz J., Gründemann D. (2018). Substrate Selectivity Check of the Ergothioneine Transporter. Drug Metab. Dispos..

[45] Suh J.H., Kim R., Yavuz B., Lee D., Lal A., Ames B.N., Mark K. (2009). Clinical Assay of Four Thiol Amino Acid Redox Couples by LC-MS/MS: Utility in Thalassemia. J. Chromatogr. B Anal. Technol. Biomed. Life Sci..

[46] Kuypers F.A. (2014). Hemoglobin S Polymerization and Red Cell Membrane Changes. Hematol. Oncol. Clin. N. Am..

[47] Halliwell B., Cheah I.K., Drum C.L. (2016). Ergothioneine, an Adaptive Antioxidant for the Protection of Injured Tissues? A Hypothesis. Biochem. Biophys. Res. Commun..

[48] Cheah I.K., Tang R., Ye P., Yew T.S.Z., Lim K.H.S., Halliwell B. (2016). Liver Ergothioneine Accumulation in a Guinea Pig Model of Non-Alcoholic Fatty Liver Disease. A Possible Mechanism of Defence?. Free Radic. Res..

[49] Tang R.M.Y., Cheah I.K.M., Yew T.S.K., Halliwell B. (2018). Distribution and Accumulation of Dietary Ergothioneine and Its Metabolites in Mouse Tissues. Sci. Rep..

[50] Cheah I.K., Halliwell B. (2020). Could Ergothioneine Aid in the Treatment of Coronavirus Patients?. Antioxidants.

[51] Schröder E., Eaton P. (2008). Hydrogen Peroxide as an Endogenous Mediator and Exogenous Tool in Cardiovascular Research: Issues and Considerations. Curr. Opin. Pharmacol..

[52] Benfeitas R., Selvaggio G., Antunes F., Coelho P.M.B.M., Salvador A. (2014). Hydrogen Peroxide Metabolism and Sensing in Human Erythrocytes: A Validated Kinetic Model and Reappraisal of the Role of Peroxiredoxin II. Free Radic. Biol. Med..

[53] Lushchak V.I. (2014). Free Radicals, Reactive Oxygen Species, Oxidative Stress and Its Classification. Chem. Biol. Interact..

[54] Lim J.B., Langford T.F., Huang B.K., Deen W.M., Sikes H.D. (2016). A Reaction-Diffusion Model of Cytosolic Hydrogen Peroxide. Free Radic. Biol. Med..

[55] Travasso R.D.M., Sampaio dos Aidos F., Bayani A., Abranches P., Salvador A. (2017). Localized Redox Relays as a Privileged Mode of Cytoplasmic Hydrogen Peroxide Signaling. Redox Biol..

[56] Chénais B., Andriollo M., Guiraud P., Belhoussine R., Jeannesson P. (2000). Oxidative Stress Involvement in Chemically Induced Differentiation of K562 Cells. Free Radic. Biol. Med..

[57] Hietakangas V., Poukkula M., Heiskanen K.M., Karvinen J.T., Sistonen L., Eriksson J.E. (2003). Erythroid Differentiation Sensitizes K562 Leukemia Cells to TRAIL-Induced Apoptosis by Downregulation of c-FLIP. Mol. Cell. Biol..

[58] Isoda H., Motojima H., Onaga S., Samet I., Villareal M.O., Han J. (2014). Analysis of the Erythroid Differentiation Effect of Flavonoid Apigenin on K562 Human Chronic Leukemia Cells. Chem. Biol. Interact..

[59] Malik Z., Chitayat S.D., Langzam Y. (1988). Hemin Dependent Morphological Maturation and Endogenous Porphyrin Synthesis by K562 Leukemic Cells. Cancer Lett..

[60] Zhang C., Guo L.Y., Mu D., Gong J.H., Chen J. (2019). Induction of Apoptosis and Erythroid Differentiation of Human Chronic Myelogenous Leukemia K562 Cells by Low Concentrations of Lidamycin. Oncol. Rep..

[61] Liang R., Menon V., Ghaffari S. (2019). Following Transcriptome to Uncover FOXO Biological Functions. Methods Mol. Biol..

[62] Menon V., Ghaffari S. (2018). Transcription Factors FOXO in the Regulation of Homeostatic Hematopoiesis. Curr. Opin. Hematol..

[63] Miyamoto K., Araki K.Y., Naka K., Arai F., Takubo K., Yamazaki S., Matsuoka S., Miyamoto T., Ito K., Ohmura M. (2007). Foxo3a Is Essential for Maintenance of the Hematopoietic Stem Cell Pool. Cell Stem Cell.

[64] Thanuthanakhun N., Nuntakarn L., Sampattavanich S., Anurathapan U., Phuphanitcharoenkun S., Pornpaiboonstid S., Borwornpinyo S., Hongeng S. (2017). Investigation of FoxO3 Dynamics during Erythroblast Development in β-Thalassemia Major. PLoS ONE.

[65] Wang H., Li Y., Wang S., Zhang Q., Zheng J., Yang Y., Qi H., Qu H., Zhang Z., Liu F. (2015). Knockdown of Transcription Factor Forkhead Box O3 (FOXO3) Suppresses Erythroid Differentiation in Human Cells and Zebrafish. Biochem. Biophys. Res. Commun..

[66] Zhang Y., Paikari A., Sumazin P., Ginter Summarell C.C., Crosby J.R., Boerwinkle E., Weiss M.J., Sheehan V.A. (2018). Metformin Induces FOXO3-Dependent Fetal Hemoglobin Production in Human Primary Erythroid Cells. Blood.

[67] Zhu X., Li B., Pace B.S. (2017). NRF2 Mediates γ-Globin Gene Regulation and Fetal Hemoglobin Induction in Human Erythroid Progenitors. Haematologica.

[68] Torres L., Conran N. (2018). Emerging Pharmacotherapeutic Approaches for the Management of Sickle Cell Disease. Expert Opin. Pharmacother..

[69] Nakamura T., Sugiura S., Kobayashi D., Yoshida K., Yabuuchi H., Aizawa S., Maeda T., Tamai I. (2007). Decreased Proliferation and Erythroid Differentiation of K562 Cells by SiRNA-Induced Depression of OCTN1 (SLC22A4) Transporter Gene. Pharm. Res..

[70] Xie W., Ma W., Liu P., Zhou F. (2019). Overview of Thioredoxin System and Targeted Therapies for Acute Leukemia. Mitochondrion.

[71] Olmos Y., Sánchez-Gómez F.J., Wild B., García-Quintans N., Cabezudo S., Lamas S., Monsalve M. (2013). SirT1 Regulation of Antioxidant Genes Is Dependent on the Formation of a FoxO3a/PGC-1α Complex. Antioxid. Redox Signal..

[72] Winterbourn C.C. (2018). Biological Production, Detection, and Fate of Hydrogen Peroxide. Antioxid. Redox Signal..

[73] Winterbourn C.C., Hampton M.B. (2015). Redox Biology: Signaling via a Peroxiredoxin Sensor. Nat. Chem. Biol..

[74] Czech M., Lawrence J., Lynn W. (1974). Hexose Transport in Isolated Brown Fat Cells. A Model System for Investigating Insulin Action on Membrane Transport. J. Biol. Chem..

[75] Mahadev K., Wu X., Zilbering A., Zhu L., Lawrence J.T.R., Goldstein B.J. (2001). Hydrogen Peroxide Generated during Cellular Insulin Stimulation Is Integral to Activation of the Distal Insulin Signaling Cascade in 3T3-L1 Adipocytes. J. Biol. Chem..

[76] Steinbrenner H. (2013). Interference of Selenium and Selenoproteins with the Insulin-Regulated Carbohydrate and Lipid Metabolism. Free Radic. Biol. Med..

[77] Szypowska A.A., Burgering B.M.T. (2011). The Peroxide Dilemma: Opposing and Mediating Insulin Action. Antioxid. Redox Signal..

[78] Guan L., Zhang L., Gong Z., Hou X., Xu Y., Feng X., Wang H., You H. (2016). FoxO3 Inactivation Promotes Human Cholangiocarcinoma Tumorigenesis and Chemoresistance through Keap1-Nrf2 Signaling. Hepatology.

[79] Dare A., Channa M.L., Nadar A. (2021). L-Ergothioneine and Its Combination with Metformin Attenuates Renal Dysfunction in Type-2 Diabetic Rat Model by Activating Nrf2 Antioxidant Pathway. Biomed. Pharmacother..

[80] Servillo L., Castaldo D., Casale R., D’Onofrio N., Giovane A., Cautela D., Balestrieri M.L. (2015). An Uncommon Redox Behavior Sheds Light on the Cellular Antioxidant Properties of Ergothioneine. Free Radic. Biol. Med..

[81] Hopkins B.L., Nadler M., Skoko J.J., Bertomeu T., Pelosi A., Shafaei P.M., Levine K., Schempf A., Pennarun B., Yang B. (2018). A Peroxidase Peroxiredoxin 1-Specific Redox Regulation of the Novel FOXO3 MicroRNA Target Let-7. Antioxid. Redox Signal..

[82] Woo H.A., Yim S.H., Shin D.H., Kang D., Yu D.Y., Rhee S.G. (2010). Inactivation of Peroxiredoxin I by Phosphorylation Allows Localized H_2_O_2_ Accumulation for Cell Signaling. Cell.

[83] Wood Z.A., Poole L.B., Karplus P.A. (2003). Peroxiredoxin Evolution and the Regulation of Hydrogen Peroxide Signaling. Science.

[84] Rhee S.G., Woo H.A. (2011). Multiple Functions of Peroxiredoxins: Peroxidases, Sensors and Regulators of the Intracellular Messenger H_2_O_2_, and Protein Chaperones. Antioxid. Redox Signal..

[85] Wang Z., Yu T., Huang P. (2016). Post-Translational Modifications of FOXO Family Proteins (Review). Mol. Med. Rep..

[86] Grune T., Reinheckel T., Davies K.J.A. (1996). Degradation of Oxidized Proteins in K562 Human Hematopoietic Cells by Proteasome. J. Biol. Chem..

[87] Takeda K., Yoshida T., Kikuchi S., Nagao K., Kokubu A., Pluskal T., Villar-Briones A., Nakamura T., Yanagida M. (2010). Synergistic Roles of the Proteasome and Autophagy for Mitochondrial Maintenance and Chronological Lifespan in Fission Yeast. Proc. Natl. Acad. Sci. USA.

[88] Morozov A.V., Karpov V.L. (2018). Biological Consequences of Structural and Functional Proteasome Diversity. Heliyon.

