# Supplementary material for: Potential Cytoprotective and Regulatory Effects of Ergothioneine on Gene Expression of Proteins Involved in Erythroid Adaptation Mechanisms and Redox Pathways in K562 Cells
Source: Genes (Basel). 2022 Dec 15;13(12):2368. doi: 10.3390/genes13122368 (PMC9778224; doi:10.3390/genes13122368)
Supplement: Supplementary file 1 [file genes-13-02368-s001.zip › genes-2010978-supplementary.pdf]

Supplementary Material

Potential Cytoprotective and Regulatory Effects of Ergothioneine on Gene Expression of Proteins Involved in Erythroid Adaptation Mechanisms and Redox Pathways in K562 Cells

Victoria Simões Bernardo [1,†], Flaviene Felix Torres [1,†], Carla Peres de Paula [2], João Pedro Maia de Oliveira da Silva [2], Eduardo Alves de Almeida [3], Anderson Ferreira da Cunha [2] and Danilo Grünig Humberto da Silva [4,*]

[1] Department of Biology, Universidade Estadual Paulista (UNESP), José do Rio Preto 15054-000, SP, Brazil
[2] Department of Genetics and Evolution, Universidade Federal de São Carlos (UFSCar), São Carlos 13565-905, SP, Brazil
[3] Department of Natural Sciences, Fundação Universidade Regional de Blumenau (FURB), Blumenau 89030-000, SC, Brazil
[4] Campus de Três Lagoas, Universidade Federal de Mato Grosso do Sul (CPTL/UFMS), Três Lagoas 79613-000, MS, Brazil
* Correspondence: danilo.grunig@ufms.br; Tel.: +55-(67)-3509-3764
† These authors contributed equally to this work.

Supplementary Figures and Table

Table S1. Primers sequences

Genes	*Forward primer*	*Reverse primer*
ACTB	5′-CAAGCAGGAGTATGACGAGTC-3′	5′- GCCATGCCAATCTCATCTTG-3′
PRDX1	5'-TGTAAATGACCTCCCTGTTGG-3'	5'-TATCACTGCCAGGTTTCCAG-3'
PRDX2	5'-CTGTTAATGATTTGCCTGTGGG -3'	5'-TGGGCTTAATCGTGTCACTG-3'
PRDX6	5'-CACGACTTTCTGGGAGACT-3'	5'-GGGCAATCAACTTAACATTCCTC-3'
CAT	5'-TGAATGAGGAACAGAGGAAACG-3'	5'-GTACTTGTCCAGAAGAGCCTG-3'
SOD1	5'-GGGCAAAGGTGGAAATGAAG-3'	5'-CAGCTAGCAGGATAACAGATGAG-3'
GPX1	5'-TTCCAGACCATTGACATCGAG-3'	5'-CACCCTCATAGATGAAAACCCC-3'
TRX1	5′- GGTGAAGCAGATCGAGAGCAAAGA-3'	5′-ACCACGTGGCTGAGAAGTCAACTA-3′
FOXO3	5′-GCGTGCCCTACTTCAAGGATAAG-3′	5′-GACCCGCATGAATCGACTATG-3′
MST1	5′-CCTCCCACATTCCGAAAACCA-3′	5′-GCACTCCTGACAAATGGGTG-3′
YWHAQ (14-3-3)	5'- GGGTTGCATCTCTTTCTTGC -3'	5′-GCACTCCTGACAAATGGGTG-3′

NRF2	5′-GCTACGTGATGAAGATGGAAAAC-3′	5′-AGCTCAGAAAAGGTCAAATCCTC-3′
KEAP1	5′- AACAGAGACGTGGACTTTCG-3′	5′- GTGTCTGTATCTGGGTCGTAAC-3′
γ-Globin	5′-TGTGGAAGATGCTGGAGGAGA-3′	5′-CAAAGAACCTCTGGGTCCATG-3′
PSMB5	5′-CCATACCTGCTAGGCACCAT-3′	5′-GCACCTCCTGAGTAGGCATC-3′
PSMB6	5′-CCTATTCACGACCGCATTTT-3′	5′-TCCCGGTAGGTAGCATCAAC-3′

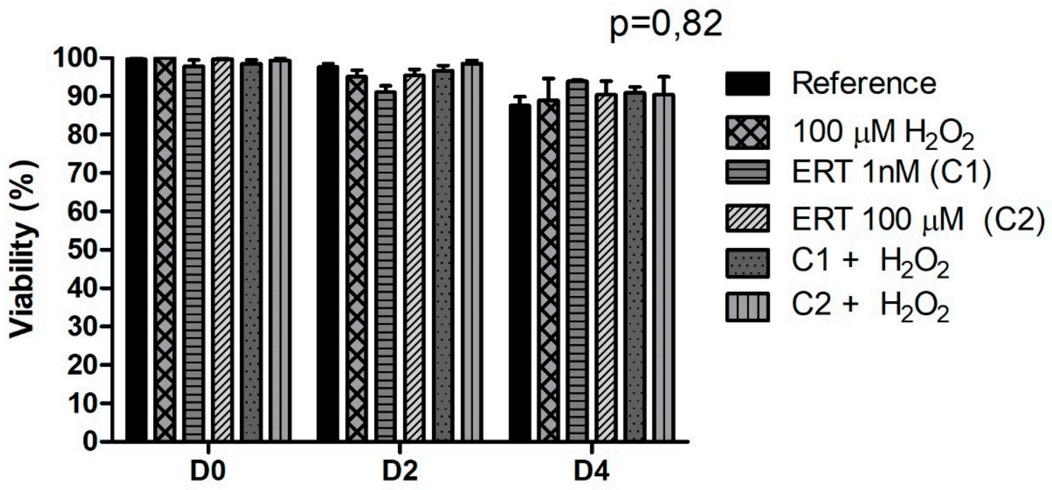

Figure S1. Viability of K562 differentiated cells under the different experimental conditions tested. Reference: K562 cells without oxidative stress induction and not treated with ERT; 100μM H_2O_2: Cells under stress induction with hydrogen peroxide; C1: cells translated with 1 nM ergothioneine (ERT); C2: cells translated with 100μM ERT; C1 + 100μM H_2O_2 and C2 + 100μM H_2O_2: sets of cells treated with the same concentrations of ERT associated with stress induction; D0: before the differentiation process, D2: beginning of cell differentiation; D4: maximum of the differentiation process. Values expressed as mean with standard error (± SEM). General Linear Models (GLM) with two-way ANOVA design.

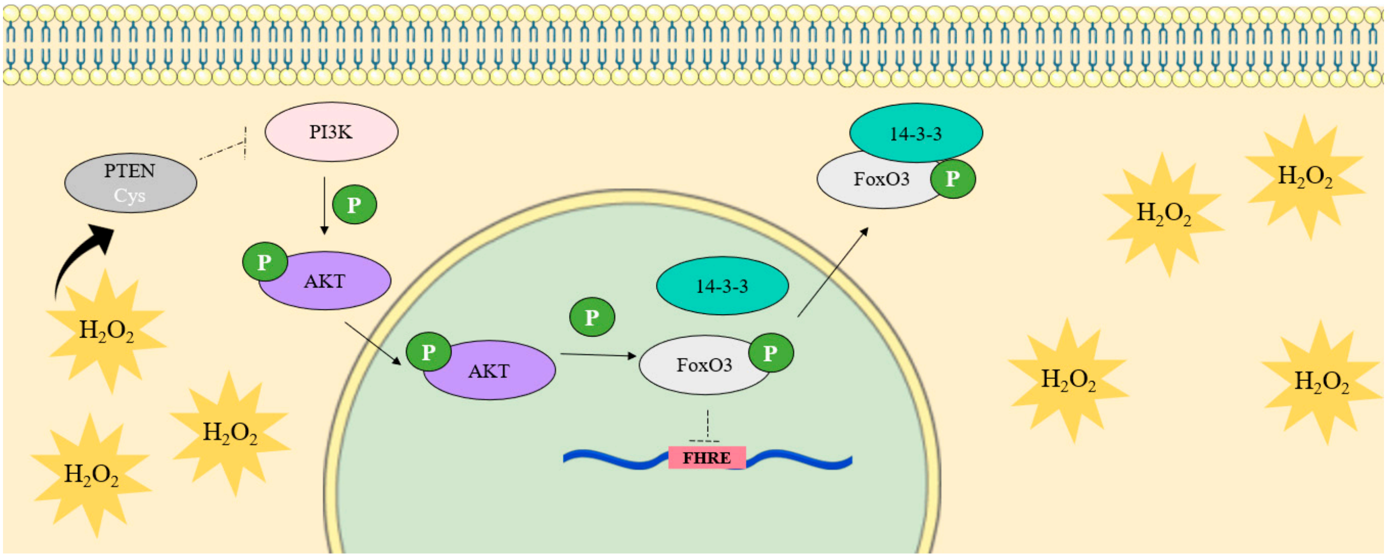

Figure S2. Cell signaling generated by hydrogen peroxide and its modulatory effect on the FoxO3 transcription factor. The hydrogen peroxide (H_2O_2) internalized by the cell generates low-intensity oxidative stress, a subtype of oxidative stress characterized by oxidation of several cellular components, such as PTEN. The oxidation of the redox-sensitive Cys residues in the active sites of this important inhibitor of PI3K causes it is transiently inactivation. This results in the negative modulation of FoxO3 thru the interrupted activation of the PI3K/AKT pathway (FoxO3 is translocated to the cytoplasm by chaperone 14-3-3). AKT: Protein kinase B; FHRE: FoxO responsive element; FoxO3, Forkhead box O protein; P: Phosphofrilation; PER: Hydrogen peroxide; PI3K: Phosphatidylinositol 3-kinase; PTEN: Phosphatase and tensin homolog on chromosome ten. Continuous line: active pathway; Dashed line: inactivated pathway. For interpretation of the references to color in this figure legend, the reader is referred to the web version of this article. Source: This figure was created by the author adapting images from Servier Medical Art Commons Attribution 3.0 Unported License (http://smart.servier.com)).

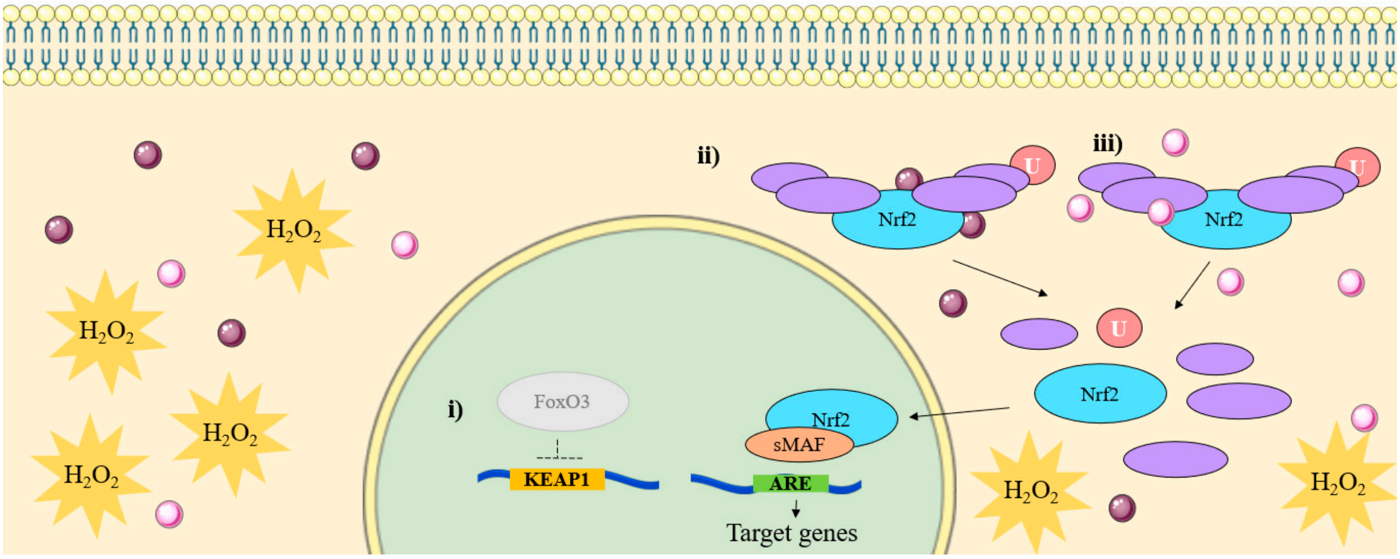

Figure S3. Proposed mechanisms of action for activating the Nrf2-ARE pathway in the treatment with the lowest concentration of ergothioneine in erythroid cells K562 under oxidative stress. The hydrogen peroxide (H_2O_2) internalized by the cell results in an oxidative intracellular environment. ERT (purple circle - reduced form; pink circle - oxidized intermediaries) is internalized by its specific ETT transporter, and acts directly, detoxifying the H_2O_2 in the cytoplasm. The activation of Nrf2 can be related to two mechanisms: i) a negative regulation of Keap1 caused by FoxO3 depletion (represented by the grayness in the FoxO3 protein); ii) ERT could bind to the Nrf2-Keap1 complex, leading to its dissociation, nuclear accumulation of the freed Nrf2, thus the activation of antioxidant genes and/or iii) the oxidized intermediaries of ERT interacts with the Keap1-Cul3-Rbx1 complex, destabilizing it and stimulates the nuclear accumulation of Nrf2. ARE: Human Antioxidant Response Element; ERT: ergothioneine; FoxO3, Forkhead box O protein; H_2O_2: hydrogen peroxide; Keap1: Kelch-like ECH-associated protein 1; Nrf2, erythroid nuclear factor 2 related to factor 2; P: phosphorylation; sMAF, Small Maf protein; U, ubiquitination. Continuous line: active pathway; Dashed line: inactive pathway. For interpretation of the references to color in this figure legend, the reader is referred to the web version of this article. Source: This figure was created by the author adapting images from Servier Medical Art Commons Attribution 3.0 Unported License (http://smart.servier.com)).
